# Fruit Antioxidants during Vinegar Processing: Changes in Content and in Vitro Bio-Accessibility

**DOI:** 10.3390/ijms17101658

**Published:** 2016-09-29

**Authors:** Sena Bakir, Gamze Toydemir, Dilek Boyacioglu, Jules Beekwilder, Esra Capanoglu

**Affiliations:** 1 Department of Food Engineering, Faculty of Chemical and Metallurgical Engineering, Istanbul Technical University, Maslak, 34469 Istanbul, Turkey; senabakir@gmail.com (S.B.); boyaci@itu.edu.tr (D.B.); 2 Department of Food Engineering, Faculty of Engineering, Recep Tayyip Erdogan University, Merkez, 53100 Rize, Turkey; 3 Department of Food Engineering, Faculty of Engineering and Architecture, Okan University, Akfirat-Tuzla, 34959 Istanbul, Turkey; toydemir.gamze@gmail.com; 4 Plant Research International, Wageningen UR, 6700 AA Wageningen, The Netherlands; jules.beekwilder@wur.nl

**Keywords:** vinegar, apple, grape, antioxidant, polyphenol

## Abstract

Background: Vinegars based on fruit juices could conserve part of the health-associated compounds present in the fruits. However, in general very limited knowledge exists on the consequences of vinegar-making on different antioxidant compounds from fruit. In this study vinegars derived from apple and grape are studied. Methods: A number of steps, starting from the fermentation of the fruit juices to the formation of the final vinegars, were studied from an industrial vinegar process. The effect of each of the vinegar processing steps on content of antioxidants, phenolic compounds and flavonoids was studied, by spectroscopic methods and by high-performance liquid chromatography (HPLC). Results: The major observation was that spectrophotometric methods indicate a strong loss of antioxidant phenolic compounds during the transition from fruit wine to fruit vinegar. A targeted HPLC analysis indicates that metabolites such as gallic acid are lost in later stages of the vinegar process. Conclusion: The major conclusion of this work is that major changes occur in phenolic compounds during vinegar making. An untargeted metabolite analysis should be used to reveal these changes in more detail. In addition, the effect of vinegar processing on bio-accessibility of phenolic compounds was investigated by mimicking the digestive tract in an in vitro set up. This study is meant to provide insight into the potential of vinegar as a source of health-related compounds from fruit.

## 1. Introduction

Fruits are known to contain a variety of health-associated compounds [1,2,3,4,5,6,7]. Among them are many vitamins and polyphenolic compounds for which part of the health effects have been associated with their antioxidant activity and are therefore referred to as anti-oxidants [8]. When tested in cell free systems or in cultures of cells, antioxidants have the ability to protect molecules such as DNA or cell walls from damage caused by free radical induced oxidative stress [9]. In dietary studies and in vivo experiments using animals, these antioxidants are associated with lower risks of degenerative diseases, particularly cardiovascular diseases and cancer besides inflammatory and neurodegenerative diseases [10,11,12,13,14,15,16]. 

In recent years, the effect of food processing on fruit antioxidants has been investigated [17,18,19,20,21,22,23]. Food processing is traditionally used to preserve vitamin sources for consumption during winter, etc. In more recent years, fruit juices have been attractive to consumers as a source for intake of beneficial compounds that occur in fruit. In order to evaluate if processed fruit products would provide the same amounts of antioxidant compounds as fresh fruits, changes in content of those antioxidants during processing should be evaluated. Moreover, in recent years it has become clear that antioxidants may differ strongly in their bio-accessibility, i.e., the fraction of each compound that is absorbed by the digestive system and is available in the blood circulation [12].

Vinegar can be made from industrial alcohol, but is often a fermentation product of fruit juices, which contains 5%–10% acetic acid. Turkey is a large producer of such fruit-based vinegars. To produce vinegar from fruit juices, a wine is produced by yeast fermentation, after which the ethanol in the wine is slowly converted to acetic acid by acetic acid bacteria. Vinegar is not only used as an acidic seasoning, but also as a specialty food ingredient, due to proclaimed beneficial effects, such as digestive effects, appetite stimulation, antioxidant effects, exhaustion-recovering effects, lipid lowering effects, and regulations of blood pressure [23,24]. 

Phenolic compounds in vinegar may derive from the starting material (i.e., the fruit), or may be introduced to it by aging of the vinegar in wooden barrels. Therefore, large differences exist in content of phenolic compounds among vinegars. For example, (+)-Catechin was not found in malt vinegar, while 8.29 mg (+)-Catechin per 100 mL of apple vinegar was observed [25]. In another case, gallic acid was not observed in alcohol vinegar [25], while others reported 9.50 mg/100 mL gallic acid in alcohol vinegar aged in wood [26]. These findings indicate that the polyphenolic composition may differ strongly between vinegars. 

The aim of this study was to determine the effect of vinegar processing on content of antioxidants, phenolic compounds and flavonoids, using spectroscopic methods. Different steps of the production process of vinegar were analyzed. In addition, the effect of vinegar processing on bio-accessibility of phenolic compounds was investigated with in vitro methods; in this way we hope to gain insight into the potential of vinegar as a source of health-related compounds.

## 2. Results

### 2.1. The Effects of Vinegar Processing on Antioxidant Compounds

As a first step, dry matter contents of grape and apple vinegar processing samples were measured, to be able to calculate the effects of processing fruit antioxidants on dry weight basis. Dry matter contents of grape and apple vinegar processing samples are given in Table 1. Subsequently, grape vinegar and apple vinegar processing samples were utilized for measuring the effects on total flavonoid content (TFC), total phenolic content (TPC) and total antioxidant capacity (TAC) of samples. Results were determined on a fresh weight basis (FWB), and calculated to dry weight basis (DWB) at tables.

#### 2.1.1. Effects of Vinegar Processing on Grape Antioxidants

The grape vinegar processing was studied for its effects on TPC, TFC, and TAC of different processing samples including while the samples collected for grape vinegar processing included grape wine (GW), raw grape vinegar (RGV), clarified grape vinegar (CGV), filtered grape vinegar (FGV), and the final packaged grape vinegar (FPGV). The TFC, TPC and TAC values, determined for grape vinegar processing samples, are represented in Table 2. From grape wine to grape vinegar, samples were not found to be significantly different regarding to their TFC and TPC when the calculations were performed on DWB (*p* > 0.05). Remarkably, samples collected from the intermediary steps of processing were all found to be significantly lower in their TFC and TPC values, in comparison to grape wine sample. The antioxidant capacity (TAC) changed also during the process of vinegar making from wine. Although statistical analysis indicates that changes are significant, the changes in activity were less than a factor 2. 

#### 2.1.2. Effects of Vinegar Processing on Apple Antioxidants

The effects of apple vinegar processing on TPC, TFC, and TAC of several processing samples was also studied. Samples include apple juice concentrate (AJC), apple wine (AW), clarified apple vinegar (CAV), filtered apple vinegar (FAV), and the final packaged apple vinegar (FPAV).

TFC, TPC and TAC of the samples are given together at Table 2. The process from apple juice concentrate to apple wine is associated with an increase in TFC and TPC. Antioxidant capacity of the samples measured with 2,2′-azino-bis(3-ethylbenzothiazoline-6-sulphonic acid) (ABTS), cupric reducing antioxidant capacity (CUPRAC), 2,2-diphenyl-1-picrylhydrazyl (DPPH) and ferric reducing antioxidant power (FRAP) showed variable results. All TAC methods show that there is a loss of TAC going from wine to vinegar in the process.

#### 2.1.3. The Effects of Vinegar Processing on Phenolic Profile

Processing effect of vinegar making on phenolic compounds were quantified using HPLC. Phenolic compounds of the grape vinegar samples were measured with HPLC-PDA and quantified (Table 3). Grape wine is rich in gallic acid, however *p*-hydroxybenzoic acid was not observed. After decantation and filtering, the gallic acid concentration sharply decreased more that 10-fold, while *p*-hydroxybenzoic acid was not detected in grape wine and raw grape vinegar, but was observed in the processing products after decantation.

Apple vinegar contains a more complex phenolic compound profile. Apart from gallic acid and *p*-hydroxybenzoic acid, also catechin, syringic acid, caffeic acid and *p*-coumaric acid were observed. Most remarkable value was observed at catechin concentration in AJC with 70.30 ± 4.90 mg/100 mL FW. The concentration of syringic acid, caffeic acid and *p*-coumaric acid declined strongly (4–10-fold) after the processing of apple juice concentrate to apple wine, while gallic acid and *p*-hydroxybenzoic acid did not show this effect.

### 2.2. The Effects of in Vitro Digestion on Vinegar Antioxidant Bio-Availability

A method for investigating in vitro bio-accessibility as described by McDougall [27] was applied to vinegars. Vinegars (“Initial” samples) were subjected to a gastric treatment (PG samples), after which serum available material (IN-samples) and material remaining in the intestinal lumen (OUT-samples) were obtained. For a detailed description see Section 4.3. On these materials, again all spectroscopic analyses—TFC, TPC and TAC—were performed (Table 4).

#### 2.2.1. The Effects of in Vitro Digestion on Grape Vınegar

Grape vinegar’s PG, IN and OUT fractions were analyzed. Modest and insignificant effects on total phenolic content (TPC) were observed (Table 4). Apparently the compounds measured in this way are not partitioned by the in vitro digestion system. The total flavonoid content (TFC) clearly dropped in the IN samples, indicating that the compounds addressed by the TFC measurements are poorly serum-available. The antioxidant capacity (TAC), addressed by different methods, showed in each methodology that the transition from initial to post-gastric fraction, and in the transition from post-gastric to serum available and non-serum available material, significant losses of activity can be observed. The recovery of antioxidants, representing the serum-available fraction of the initial material, is 10% to 40%, depending on the analytical method used. 

#### 2.2.2. The Effects of in Vitro Digestion on Apple Vinegar

Similar to grape vinegar, apple vinegar showed losses of antioxidant activities during in vitro digestion, ranging from 11% to 44% recovery in the serum available fraction (IN), depending on the used method.

#### 2.2.3. The Effects of in Vitro Bio-Accessibility on Vinegar Phenolic Profile

When gallic acid and *p*-hydroxybenzoic acid were followed during in vitro digestion using high-performance liquid chromatography (HPLC), losses and low recovery numbers were obtained: in particular *p*-hydroxybenzoic acid is recovered to less than 10%. Gallic acid is recovered slightly better, but still with <20% efficiency (Table 5). Both gastric treatment (PG vs. initial) and pancreatic treatment (IN and OUT vs. PG) have a strong effect, while IN and OUT show similar numbers, indicating that both compounds diffuse freely over the membrane. Numbers are rather similar for both types of vinegar, indicating that the matrix of these compounds has a limited effect on their in vitro bioaccessibility.

## 3. Discussion

This study was meant to provide an initial view on processes occurring during vinegar-making, from the perspective of phenolic antioxidants. Two different sorts of analytical methodology were tested: a number of spectroscopic assays, which are aimed to provide a more global impression of effects on phenolic compounds, and a dedicated HPLC method, which is largely focused on specific phenolic compounds which are known to occur in vinegar. 

The outcome from this study is that the spectroscopic studies are not straightforward to interpret. For example, some antioxidant activity assays like the ABTS assay show almost no change in value between apple juice concentrate and apple wine samples (Table 2), while the CUPRAC method for measuring antioxidant activity reports an almost 2-fold increase in activity in the apple wine samples, compared to the juice. Each of these methods measures a different aspect of antioxidant activity, but could, for example, also respond differently to acidity values or ethanol content of samples [28], and thus change values irrespective of the content of phenolic compounds. Niki [29] also points out that the capacity of free radical scavenging does not always correlate well with the capacity to inhibit oxidation. The dedicated method is much more accurate, in that it is much less sensitive to the matrix of the specific phenolic compounds, while a drawback of this method is that it only focusses on a few compounds, while compounds may undergo a transition during the making of vinegar (polymerization, partial oxidation) which not necessarily completely abolishes their relevant bioactivity. For example, this can be observed in the occurrence of *p*-hydroxybenzoic acid in grape vinegar only after the decanting of vinegar, while the gallic acid content of vinegar sharply declines after decantation. Given the highly related structure of both compounds, one could expect that *p*-hydroxybenzoic acid is formed along similar lines as gallic acid, for example by oxidation of more complex, possibly polymeric phenolic compounds [30]. Therefore both methods have their virtues and their drawbacks. More comprehensive analysis of the metabolite changes during fruit processing, using untargeted HPLC methods coupled to mass spectrometry (HPLC-MS), has been performed for the processing of tomato fruit to tomato paste [17], and for the processing of sour cherry fruit to fruit juice [20]. Given the great changes observed for the phenolic compounds identified in grape and apple vinegar (Table 3), such an untargeted HPLC-MS analysis would reveal many more changes than are now already disclosed by the dedicated HPLC analysis.

A number of studies have described the antioxidant properties of vinegars [26,31,32,33]. A more detailed analysis was presented by Ubeda et al. [34], who focused on the effect of different processing techniques on antioxidant activity and total phenols during a laboratory production of persimmon vinegars. In their analysis, hardly any changes were observed in antioxidant activity during vinegar production. This contrasts with the findings in our study on commercial apple- and grape-vinegar production (Table 1), where, depending on the method used, loss of antioxidant capacity up to a factor 2 is observed. 

Cerezo et al. [35] studied the anthocyanin composition and antioxidant activity of Cabernet Sauvignon red wine vinegar produced by submerged acidification in wood. Antioxidant activity of wine and vinegar was determined as 11.23 ± 0.11 and 9.61 ± 0.20 mM Trolox equivalent antioxidant capacity (TEAC)/g sample extract for FRAP essay, 12.26 ± 0.18 and 10.2 ± 0.3 mM TEAC/g sample extract for DPPH essay. A decrease in antioxidant values from wine to vinegar by 14.4%, 16.8% for FRAP and DPPH tests was observed. These losses in antioxidant values from wine to vinegar are slightly lower than those observed in our study. This difference is possibly due to utilization of different red grape varieties, and the use of wooden barrels for vinegar production.

The bio-accessibility of phenolic antioxidants from vinegar is a matter of great interest, and in this work we try to develop methods to address it. From previous studies it has become clear that the presence of matrix compounds such as glucose and citric acid can have a major impact on the passage of polyphenols over intestinal epithelial cells [27,36] and in an in vitro gastrointestinal system such as the system described in this work [21]. In addition to these, bioavailability of antioxidants depends on several factors such as the antioxidant related factors (i.e., chemical structure, molecular linkage), the food matrix and the processing method used, which need further research [37]. In follow-up research of the work on vinegars, the effect of matrix compounds such as acetic acid on the bio-accessibility of vinegar phenolic compounds like gallic acid should be studied.

## 4. Materials and Methods

### 4.1. Vinegar Samples

The vinegar processing samples from apples (*Malus domestica*) and grapes (*Vitis vinifera*) were supplied by the Kühne Turkey Vinegar Factory (İzmir, Turkey), from at least three independent samplings. In processing of both types of vinegar, the fruit juice concentrate, of the respective vinegars, with an average of 60%–70% dry matter content, is first subjected to an alcoholic fermentation, via the use of yeast (*Saccharomyces cerevisiae*), until the ethyl alcohol content reaches to 8%–12% (*v*/*v*) for grape and 9%–11% (*v*/*v*) for apple, and this intermediary product is termed as “wine”. Subsequently, the acetous fermentation of ethanol into acetic acid takes place with the use of acetic acid bacteria, and the resulting product, with an acetic acid concentration of ≈10%, is called as “raw vinegar”. Raw vinegar was stored for 3 months, in resin-coated polyester tanks, which provides maturation for the development of the acceptable aroma and quality. When maturation is completed, clarification process takes place by application of bentonite, kieselghur and gelatin for 1–2 days. Following the clarification step, filtration step is carried out, for 4 h, in which the cross-flow microfiltration is applied with the use of ceramic membranes with a pore size of 0.2 μm. This “filtered vinegar” with an acetic acid concentration of 10% is diluted to an acid concentration of 4% by adding water at packaging step. At this packaging step, the vinegar is lastly supplemented with 1–2 mg of sodium metabisulfite in order to get the “final vinegar” product. 

Apple and grape vinegar processing samples were collected from different steps of above-mentioned vinegar processing protocol. The samples collected for apple vinegar processing were “apple juice concentrate” (AJC), “apple wine” (AW), “clarified apple vinegar” (CAV), “filtered apple vinegar” (FAV), and the “final packaged apple vinegar” (FPAV); while the samples collected for grape vinegar processing included “grape wine” (GW), “raw grape vinegar” (RGV), clarified grape vinegar” (CGV), “filtered grape vinegar” (FGV), and the “final packaged grape vinegar” (FPGV). Samples were stored at room temperature and just before analysis, all vinegar samples were centrifuged at 2700× *g* for 4 min to eliminate the haze-causing compounds. Experiments were performed in triplicate and the results were given as the mean values ± standard deviations for these triplicate measurements.

### 4.2. Dry Matter Content

Vinegar processing samples were analysed for their Brix values, using an Abbemat Refractometer (Anton Paar, Graz, Austria) at room temperature, in order for the conversion of fresh-weight basis results to dry-weight basis results. 

### 4.3. In Vitro Bioaccessibility Method

The in vitro bioaccessibility method was adapted from a study of McDougall et al. [27]. The preparation steps include firstly a solution of 0.05 g pepsin in 50 mL of 0.1 M of HCl. Approximately 37.5 mL of this solvent was taken into a flask and 1 g NaCl was added and total volume was adjusted to 500 mL with distilled water, in order to prepare stomach solvent. For preparing small intestinal media, 10.5 g of NaHCO_3_ was adjusted 250 mL with distilled water. 20 mL of this solution was taken into a dialysis bag of 20 cm length and both its ends were closed. Finally, 0.1 g of pancreatin and 0.625 g of bile salt were dissolved in 25 mL distilled water separately and then mixed with each other, and used as pancreatin-bile salts mixture. 

To mimic the digestive system, approximately 5 mL of samples were taken into a 250 mL beaker. The total volume was adjusted to 20 mL with stomach solution. Mixture was shaken for homogenous dispersion for 10 s, and pH was set to 2.0 ± 0.5 with 5 N HCl. Subsequently, samples were placed into shaker water bath for 2 h and 100 rpm at 37 °C and at the end of this period, 2 mL of the material was taken from the beaker as “Post Gastric Solutin (PG)”. The NaHCO_3_ dialysis bag was put into the beaker with the remaining post-gastric solution and 4.5 mL of pancreatin-bile salt mixture was added. The beaker was placed into the shaker water bath for 2 h at 37 °C again. After this step, a sample was drawn from inside the dialysis bag, and was called as “IN fraction”, and a sample taken outside the dialysis bag was called as “OUT fraction”. Those fractions were taken into eppendorf tubes and centrifuged at 14,000× *g*, 4 °C.

### 4.4. Determination of Total Phenolic and Flavonoid Content of Vinegars

#### 4.4.1. Total Phenolic Content

Total phenolic content (TPC) of samples was measured based on Folin-Ciocalteu method [38]. Briefly, 100 μL of sample was put into an analyis tube and 900 μL water was added. Subsequently 5 mL of 0.2 N of Folin-Ciocalteau reagent was added and incubated for 3 min. Then, 4 mL of saturated Na_2_CO_3_ solvent was added and the mixture was incubated for 90 min. At the end of this period absorbance was measured at 765 nm against a blank by using a spectrophotometer (Shimadzu UV-1700; Shimadzu Corporation, Kyoto, Japan). Gallic acid, 0.01–0.6 mg/mL in 75% MeOH, was used for generating the standard curve.

#### 4.4.2. Total Flavonoid Content

Total flavonoid content (TFC) was determined based on [39]. In brief, 250 μL of sample was taken into an analysis tube and 1.25 mL distilled water was added to sample. Afterwards, 75 μL of 5% NaNO_2_ solvent was added and the mixture was kept for 6 min, then 150 μL of 10% AlCl_3_·6H_2_O solution was added. After 5 min, 0.5 mL of 1 M NaOH was added and the total volume was adjusted to 2.5 mL with distilled water. Absorbance was measured at 510 nm wavelength against a blank. Experiments were conducted in triplicate and mean values were reported. Catechin, 0.01–0.5 mg/mL in 75% MeOH, was used for generating the standard curve.

### 4.5. Determination of Total Antioxidant Capacity

Measurements of total antioxidant capacity (TAC) of vinegar samples were performed using four different methods, which are generally used for fruits and vegetables. Experiments were conducted in triplicate and mean values were reported. Trolox (6-hydroxy-2,5,7,8-tetramethylchroman-2-carboxylic acid), 0.01–0.8 mg/mL in 75% MeOH, was used for the standard curve.

#### 4.5.1. 2,2′-Azino-bis(3-ethylbenzothiazoline-6-sulphonic acid) (ABTS) Method

The ABTS method used was based on [40]. Briefly, 220 mg of 2,2′-azino-bis(3-ethylbenzothiazoline-6-sulphonic acid) (ABTS•) was dissolved in 200 mL of distilled water and 38 mg of K_2_S_2_O_8_ was dissolved in 2 mL of distilled water. These solutions were mixed and stored overnight in the dark to complete the radicalization. After this process, ABTS• solution was obtained. ABTS• solution was diluted with 0.05 M KPi buffer (pH = 8) until its absorbance reached to the values of 0.9 ± 0.2 at 734 nm. Approximately 100 μL of sample was taken into an analysis tube and 1 mL of ABTS• solution was added to sample with the mixture was vortexed for 15 s. Absorbance was measured after 45 s at 734 nm wavelength against water. 

#### 4.5.2. DPPH Method

The 2,2-diphenyl-1-picrylhydrazyl (DPPH) method was based on [41]. In brief, 2 mL of 0.1 mM DPPH was mixed with 100 µL of sample in a test tube. Samples were stored in the dark at room temperature for 30 min. Absorbance was measured at 517 nm against methanol. 

#### 4.5.3. Cupric Reducing Antioxidant Capacity (CUPRAC) Method

The cupric reducing antioxidant capacity (CUPRAC) method was based on [42]. Briefly, 0.4262 g of CuCl_2_·2H_2_O was dissolved in 250 mL of distilled water, 19.27 g of NH_4_Ac was diluted in 250 mL of distilled water, and 0.039 g of Neocuproine was dissolved in 96% EtOH and diluted to 25 mL. Approximately 100 μL of sample was taken into an analysis tube and 1 mL of CuCl_2_·2H_2_O solvent, 1 mL of Neocuproine, 1 mL of NH_4_Ac buffer and 1 mL of distilled water were added sequentially. After incubating the mixture for 30 min, absorbance was measured at 450 nm against a blank.

#### 4.5.4. Ferric Reducing Antioxidant Power (FRAP) Method

The ferric reducing antioxidant power (FRAP) method was adapted from [43]. Briefly, 3.1 g of CH_3_COONa·3H_2_O was dissolved in distilled water, 16 mL of 99.85% acetic acid was added and the total volume was adjusted to 1 L with distilled water. 0.504 g of FeCl_3_·6H_2_O was dissolved in distilled water and mixed with 1 M HCl. The total volume of the mixture was adjusted to 100 mL with distilled water. 0.156 g of tripyridyl triazine (TPTZ) was dissolved in 50 mL ethanol. FRAP reagent was prepared with 10:1:1 volume rate with these solution sequence. Afterwards, 100 µL of sample was taken into a test tube and 900 µL of FRAP reagent was added. After keeping the mixture for 4 min at room temperature, absorbance was measured at 593 nm against distilled water.

### 4.6. HPLC Analysis of Vinegar Phenolic Profile

Phenolic profiles of all samples were also evaluated by HPLC coupled to a photodiode array (HPLC-PDA). Phenolic compounds that have been reported in vinegars, such as gallic acid, protocatechuic acid, *p*-hydroxybenzoic acid, (+)-catechin, syringic acid, caffeic acid, *p*-coumaric acid, were determined [44,45,46,47]. HPLC-PDA results of samples were given as mg/100 mL samples for all. HPLC analysis were carried out by using the method adapted from [10]. Standard calibration curves were prepared by using gallic acid, protocatechuic acid, pHBA(*p*-hydroxy benzoic acid), caffeic acid, vanilic acid, (+)-catechin, *p*-coumaric acid and syringic acid. These samples and stock solutions were filtered through a 0.45-µm membrane filter and 1 mL of the filtered sample was placed into vials and analyzed in a Waters W600 HPLC system with PDA (Waters 996) detector, for each sample. Luna C18 column (Phenomenex, Utrecht, The Netherlands), heated to 40 °C, was used as the stationary phase.

The mobile phase included solvent A (distilled water with 0.1% (*v*/*v*) trifluoric acid (TFA)) and solvent B (acetonitrile with 0.1% (*v*/*v*) TFA), acetonitrile with 0.1% (*v*/*v*) TFA. A linear gradient was used as follows: at 0 min, 95% solvent A and 5% solvent B; at 45 min, 65% solvent A and 35% solvent B; at 47 min, 25% solvent A and 75% solvent B; and at 54 min returns to initial conditions. The flow rate was 1 mL/min. Chromatograms were recorded at 280, 312, 360, and 520 nm. Identification was based on the retention times and characteristic UV spectra and quantification was done by external standard curves. 

### 4.7. Statistical Analyses

The results were analyzed by SPSS Statistics Program (21th version, IBM, New York, NY, USA) by using one way analysis of variance (ANOVA) at 0.05 significance level, and Tukey’s New Multiple Range Test was applied for post hoc tests. The differences between all samples, PG, IN and OUT fractions were evaluated statistically. Tukey’s Range Test was applied to exact values to observe the differences between TFC, TPC and TAC (*p* < 0.05). Each analysis was performed in triplicate and the results were reported as mean value ± standard deviation.

## 5. Conclusions

From this work we conclude that a number of relevant antioxidant compounds that derive from the fruits are present in apple and grape vinegar. The initial results, including dedicated analytical methods and spectroscopic methods, indicate that the processing from fruit to vinegar has a strong effect on the presence of these compounds. This warrants a further study into the more global effects of processing. Also the in vitro digestion and bio-accessibility studies indicate that phenolic vinegar compounds undergo major reductions during food intake, indicating that there could be an interaction between vinegar processing and bio-accessibility.

## Figures and Tables

**Table 1:** The percent dry matter values for the apple and grape vinegar processing samples.

Apple Vinegar Processing		Grape Vinegar Processing	
Samples	% Dry Matter	Samples	% Dry Matter
AJC	70.50 ± 0.01	GW	7.50 ± 0.01
AW	7.50 ± 0.01	RGV	8.25 ± 0.01
CAV	8.8 ± 0.1	CGV	7.8 ± 0.1
FAV	7.8 ± 0.1	FGV	8 ± 1
FPAV	4.3 ± 0.4	FPGV	3.8 ± 0.3

Results are given in percent dry matter. GW is grape wine; RGV is raw grape vinegar (not decanted and not filtered); CGV is clarified grape vinegar; FPGV is final packaged grape vinegar; and FGV is final grape vinegar; AJC is apple juice concentrate; AW is apple wine; CAV is clarified apple vinegar; FPAV is final packaged apple vinegar; FAV is final apple vinegar product.

**Table 2:** Total flavonoid, phenolic content, and total antioxidant capacity of grape and apple vinegar processing samples.

Samples		TFC ^x^	TPC ^y^	ABTS ^z^	CUPRAC ^z^	DPPH ^z^	FRAP ^z^
Grape Vinegar	GW	145 ± 7 ^a^	918 ± 144 ^a^	1495 ± 328 ^a^	1577 ± 182 ^b^	1410 ± 25 ^b^	433 ± 24 ^b^
	RGV	79 ± 30 ^b^	676 ± 69 ^b^	1062 ± 234 ^a^	1138 ± 206 ^c^	902 ± 31 ^c^	341 ± 14 ^c^
	CGV	81 ± 14 ^b^	492 ± 27 ^c^	418 ± 49 ^b^	1024 ± 99 ^c^	870 ± 44 ^c^	293 ± 23 ^c^
	FGV	101 ± 16 ^b^	506 ± 37 ^c^	441 ± 36 ^b^	1042 ± 164 ^c^	888 ± 39 ^c^	302 ± 29 ^c^
	FPGV	153 ± 19 ^a^	842 ± 171 ^a^	1158 ± 207 ^a,b^	2048 ± 191 ^a^	1612 ± 244 ^a^	568 ± 76 ^a^
Apple Vinegar	AJC	47 ± 4 ^c^	232 ± 19 ^d^	2561 ± 260 ^a^	831 ± 106 ^b^	1470 ± 74 ^a^	321 ± 20 ^b^
	AW	240 ± 10 ^a^	519 ± 55 ^a^	2372 ± 224 ^a^	1602 ± 124 ^a^	1571 ± 111 ^a^	537 ± 16 ^a^
	CAV	81 ± 12 ^b^	383 ± 12 ^c^	570 ± 49 ^c^	648 ± 105 ^c^	712 ± 28 ^c^	257 ± 16 ^c^
	FAV	64 ± 14 ^b^	357 ± 21 ^c^	587 ± 21 ^c^	565 ± 111 ^c^	714 ± 22 ^c^	254 ± 8 ^c^
	FPAV	42 ± 9 ^c^	459 ± 58 ^b^	1256 ± 110 ^b^	908 ± 118 ^b^	1087 ± 149 ^b^	323 ± 31 ^b^

^x^: mg catechin equivalente (CA)/100 mg samples at DWB; ^y^: mg gallic acid equivalents (GAE)/100 mg samples at DWB; ^z^: mg Trolox equivalent antioxidant capacity (TEAC)/100 mL at fresh weight basis (FWB). GW is grape wine; RGV is raw grape vinegar (not decanted and not filtered); CGV is clarified grape vinegar; FPGV is final packaged grape vinegar; and FGV is final grape vinegar; AJC is apple juice concentrate; AW is apple wine; CAV is clarified apple vinegar; FPAV is final packaged apple vinegar; FAV is final apple vinegar product; ABTS is 2,2′-azino-bis(3-ethylbenzothiazoline-6-sulphonic acid); CUPRAC is cupric reducing antioxidant capacity; DPPH is 2,2-diphenyl-1-picrylhydrazyl; FRAP is ferric reducing antioxidant power. Different lowercase letters (a, b, c and d) indicate statistically significant difference (*p* < 0.05).

**Table 3:** Phenolic acid profiles of the grape and apple vinegar processing samples.

Samples		Gallic Acid	*p*-Hydroxybenzoic Acid	Catechin	Syringic Acid	Caffeic Acid	*p*-Coumaric Acid
Grape Vinegar	GW	67 ± 24	N.D.	N.D.	N.D.	N.D.	N.D.
	RGV	63 ± 1	N.D.	N.D.	N.D.	N.D.	N.D.
	DGV	5 ± 2	1.1 ± 0.5	N.D.	N.D.	N.D.	N.D.
	DFGV	5 ± 2	1 ± 1	N.D.	N.D.	N.D.	N.D.
	FGV	6 ± 2	0.90 ± 0.05	N.D.	N.D.	N.D.	N.D.
Apple Vinegar	AJC	1.0 ± 0.1	1.8 ± 0.1	70 ± 5	5.2 ± 0.3	10.3 ± 0.1	2.0 ± 0.1
	AW	0.22 ± 0.02	0.20 ± 0.02	16 ± 1	0.48 ± 0.03	2.3 ± 0.1	0.30 ± 0.01
	DAV	1.8 ± 0.1	1.1 ± 0.3	7.4 ± 0.2	0.4 ± 0.1	1.12 ± 0.04	0.20 ± 0.02
	DFAV	5.2 ± 0.1	1.2 ± 0.1	2.8 ± 0.3	0.20 ± 0.01	0.52 ± 0.01	0.11 ± 0.01
	FAV	0.8 ± 0.4	0.2 ± 0.1	2.4 ± 0.1	0.12 ± 0.02	0.40 ± 0.01	0.08 ± 0.01

Results are given in mg/100 mL sample. N.D.: Not detected; GW is grape wine; RGV is raw grape vinegar (not decanted and not filtered); DGV is decanted grape vinegar; DFGV is decanted and filtered grape vinegar; and FGV is final grape vinegar; AJC is apple juice concentrate; AW is apple wine; DAV is decanted apple vinegar; DFAV is decanted and filtered apple vinegar; FAV is final apple vinegar product.

**Table 4:** Total flavonoid, phenolic content, and total antioxidant capacity of in vitro digested grape and apple vinegars.

Samples		TFC *	TPC **	ABTS ***	CUPRAC ***	DPPH ***	FRAP ***
Grape Vinegar	INITIAL	5.5 ± 0.1 ^a^	26 ± 1 ^a^	56 ± 6 ^a^	58 ± 11 ^a^	28 ± 3 ^a^	42 ± 4 ^a^
	PG	4.6 ± 0.2 ^b^	25 ± 4 ^a^	31.3 ± 0.3 ^b^	46 ± 7 ^b^	10 ± 2 ^b^	24 ± 1 ^b^
	IN	2.05 ± 0.07 ^c^	22 ± 5 ^a^	6 ± 1 ^c^	23 ± 3 ^c^	5 ± 1 ^c^	5 ± 1 ^d^
	OUT	4.0 ± 0.2 ^c^	22 ± 3 ^a^	7.5 ± 0.4 ^c^	25 ± 4 ^c^	6 ± 2 ^c^	15 ± 1 ^c^
	Recovery %	37.1	83.6	10.6	39.6	18.5	12.5
Apple Vinegar	INITIAL	2 ± 1 ^b^	17 ± 1 ^a^	37 ± 7 ^a^	48 ± 8 ^a^	13 ± 1 ^a^	30 ± 4 ^a^
	PG	3.7 ± 0.2 ^a^	13 ± 2 ^b^	24 ± 1 ^b^	29 ± 3 ^b^	7 ± 4 ^b^	13 ± 2 ^b^
	IN	2.4 ± 0.2 ^b^	12 ± 2 ^b^	8 ± 2 ^c^	21 ± 2 ^c^	3 ± 1 ^c^	3 ± 1 ^c^
	OUT	2.9 ± 0.3 ^b^	16 ± 4 ^a,b^	5.9 ± 0.4 ^c^	15 ± 5 ^c^	5 ± 2 ^b,c^	5 ± 1 ^c^
	Recovery %	100.8	73.8	21.6	43.6	23.0	11.3

*: mg CA/100 mL sample; **: mg GAE/100 mL sample; ***: mg TEAC/100 mL sample. GW is Grape wine; RGV is raw grape vinegar (not decanted and not filtered); DGV is decanted grape vinegar; DFGV is decanted and filtered grape vinegar; and FGV is final grape vinegar; AJC is apple juice concentrate; AW is apple wine; DAV is decanted apple vinegar; DFAV is decanted and filtered apple vinegar; FAV is final apple vinegar product; PG is post gastric solution; IN is solution inside the dialysis tube; OUT is solution outside the dialysis tube. Different lowercase letters (a, b, c and d) indicate statistically significant difference (*p* < 0.05).

**Table 5:** The phenolic acid profiles of the in vitro digested grape and apple vinegars.

Samples		Gallic Acid	*p*-Hydroxybenzoic Acid
Grape Vinegar	INITIAL	5.2 ± 0.2 *	0.45 ± 0.01
	PG	1.63 ± 0.01	0.1 ± 0.1
	IN	0.66 ± 0.01	0.03 ± 0.01
	OUT	0.77 ± 0.04	0.010 ± 0.001
	Recovery %	12.8	6.7
Apple Vinegar	INITIAL	0.92 ± 0.05	0.34 ± 0.05
	PG	0.31 ± 0.01	0.09 ± 0.01
	IN	0.18 ± 0.01	0.03 ± 0.01
	OUT	0.21 ± 0.01	0.02 ± 0.01
	Recovery %	19.6	8.8

* Results are given in mg/100 mL sample.

## Abbreviations

AJC	Apple Juice Concentrate
AW	Apple Wine
DAV	Decanted Apple Vinegar
DFAV	Decanted and filtered apple vinegar
DFGV	Decanted and filtered grape vinegar
DGV	Decanted Grape Vinegar
FAV	Final Apple Vinegar Product
FGV	Final Grape Vinegar Product
GW	Grape Wine
RGV	Raw Grape Vinegar
TAC	Total Antioxidant Capacity
TFC	Total Flavonoid Content
TPC	Total Phenolic Content
ABTS	2,2′-azino-bis(3-ethylbenzothiazoline-6-sulphonic acid)
DPPH	2,2-diphenyl-1-picrylhydrazyl
CUPRAC	Cupric reducing antioxidant capacity
Trolox	6-hydroxy-2,5,7,8-tetramethylchroman-2-carboxylic acid
FRAP	Ferric reducing antioxidant power
TPTZ	Tripyridyl triazine
TEAC	Trolox equivalent antioxidant capacity
ANOVA	Analysis of variance
